# Automated Detection Framework of the Calcified Plaque with Acoustic Shadowing in IVUS Images

**DOI:** 10.1371/journal.pone.0109997

**Published:** 2014-11-05

**Authors:** Zhifan Gao, Wei Guo, Xin Liu, Wenhua Huang, Heye Zhang, Ning Tan, William Kongto Hau, Yuan-Ting Zhang, Huafeng Liu

**Affiliations:** 1 Shenzhen College of Advanced Technology, University of Chinese Academy of Sciences, Shenzhen, China; 2 Shenzhen Institutes of Advanced Technology, Chinese Academy of Sciences, Shenzhen, China; 3 Key Laboratory of Biomedical information and Health Engineering, Chinese Academy of Sciences, Shenzhen, China; 4 Department of Cardiology, Guangdong Cardiovascular Institute, Guangdong General Hospital, Guangdong Academy of Medical Sciences, Guangzhou, China; 5 Institute of Clinical Anatomy, Southern Medical University, Guangzhou, China; 6 Institute of Cardiovascular Medicine and Research, LiKaShing Faculty of Medicine, University of Hong Kong, Hong Kong, China; 7 The Joint Research Centre for Biomedical Engineering, Department of Electronic Engineering, Chinese University of Hong Kong, Hong Kong, China; 8 State Key Laboratory of Modern Optical Instrumentation, Department of Optical Engineering, Zhejiang University, Hangzhou, China; College of Mechatronics and Automation, National University of Defense Technology, China

## Abstract

Intravascular Ultrasound (IVUS) is one ultrasonic imaging technology to acquire vascular cross-sectional images for the visualization of the inner vessel structure. This technique has been widely used for the diagnosis and treatment of coronary artery diseases. The detection of the calcified plaque with acoustic shadowing in IVUS images plays a vital role in the quantitative analysis of atheromatous plaques. The conventional method of the calcium detection is manual drawing by the doctors. However, it is very time-consuming, and with high inter-observer and intra-observer variability between different doctors. Therefore, the computer-aided detection of the calcified plaque is highly desired. In this paper, an automated method is proposed to detect the calcified plaque with acoustic shadowing in IVUS images by the Rayleigh mixture model, the Markov random field, the graph searching method and the prior knowledge about the calcified plaque. The performance of our method was evaluated over 996 in-vivo IVUS images acquired from eight patients, and the detected calcified plaques are compared with manually detected calcified plaques by one cardiology doctor. The experimental results are quantitatively analyzed separately by three evaluation methods, the test of the sensitivity and specificity, the linear regression and the Bland-Altman analysis. The first method is used to evaluate the ability to distinguish between IVUS images with and without the calcified plaque, and the latter two methods can respectively measure the correlation and the agreement between our results and manual drawing results for locating the calcified plaque in the IVUS image. High sensitivity (94.68%) and specificity (95.82%), good correlation and agreement (>96.82% results fall within the 95% confidence interval in the Student t-test) demonstrate the effectiveness of the proposed method in the detection of the calcified plaque with acoustic shadowing in IVUS images.

## Introduction

Atherosclerosis is the process of artery wall thickness [1], which is caused by fatty substances, cholesterol, cellular waste products, calcium and fibrin (a clotting material in the blood) building up in the inner lining of an artery, and the result of such continuous building is called plaque. The atherosclerotic plaque in coronary arteries [2] can be vital, because it can evolve toward clinical complications like angina, myocardial infarction, sudden cardiac death. The traditional imaging modality to diagnose atherosclerosis is angiography (first developed by 1927), which can visualize the longitudinal sectional image of vessels. However, angiography has several disadvantages: (1) the injury of human body caused by X-ray radiation; (2) the difficulty in distinguishing the vessels with or without the positive remodeling; (3) the diagnosis in reliance on the observer's experiences.

Recently, intravascular ultrasound (IVUS) has been widely adopted by the clinical doctor for better diagnosis of the arteriosclerotic vascular disease [3], [4]. It is an invasive imaging technology that can present the real-time visualization of vessel morphology in vivo, through high-resolution image sequences of the vascular internal structure including lumen, intima, adventitia, plaque components, vessel side-branchings etc. In order to better diagnose the status of atherosclerosis, the doctor usually draws the lumen, the vessel wall, and the plaque components manually. However, the manual analysis is a very time-consuming process and can easily lead to the inter- and intra-observer variability.

Therefore, it is highly desired to develop the computer-aided algorithm for analyzing IVUS images in order to increase diagnostic efficiency since well-designed computer-aided analysis could generate more objective conclusions. One important application of the computer-aided analysis in IVUS images is the determination of the stenosis degree and the area of blood flows inside the lumen by detecting the lumen border and the media-adventitia border. A large number of computer-aided algorithms have been developed in this area. Most of these algorithms were implemented based on the image descriptor and the contour evolution: (1) among the image descriptor based algorithms, the gradient is one widely used image descriptor [5]. However, it is susceptible to speckle noise and varieties of artifacts in IVUS, such as stents and the guide wire. Therefore, some other image descriptors, such as edge pattern [6], textural information [7], [8] and statistical properties of the pixel intensity [9], [10], have been proposed to overcome the disadvantages of the gradient descriptor; (2) in contour evolution based algorithms, the vessel borders are usually detected by evolving the per-defined initial contour until convergence. These algorithms could be divided into snake-based [4], [11] and level-set-based algorithms [12], [13]. The snake-based algorithms always treat the evolving contour as a parametric curve, and compute the desired border by minimizing the curve energy. Unlike the snake-based algorithms, the level-set-based algorithms often apply implicit functions to format the contour and evolve the contour of the zero level by solving the partial differential equations [14]. Another important application of the computer-aided analysis of IVUS images is the detection of the calcified plaque with acoustic shadowing. In the clinical expert consensus documents from the American College of Cardiology and the American Heart Association [15], [16], the calcified plaque can be used to detect obstructive coronary artery disease, and moreover it can also be considered as an accurate indicator of atherosclerotic disease and the risk of acute myocardial infarction [17], especially in diabetics [18], smokers [19] and elderly [20]. The methods for detecting the calcified plaque with acoustic shadowing could be divided into two categories: (1) the first kind of approaches will look for the calcified plaque inside the region between the lumen and the vessel wall, after the lumen border and the media-adventitia border are both detected. Because the identification of these two borders could narrow down the region of interest (ROI) for facilitating the detection of the calcified plaque, some classification algorithms have been used to extract the calcified plaque from the ROI, such as Bayesian classifier [13] or fuzzy k-means [21]. However, the performance of these classification algorithms relies on the accurate location of the lumen and media-adventitia borders, and the calcified plaque with acoustic shadowing can likely damage the accuracy because it can be considered as an artifact to obstruct the detection of the two vessel borders. For the media-adventitia border, it may be shaded by the acoustic shadowing because the most of the ultrasonic wave can be reflected by the calcified plaque, and for the lumen border, it may be represented as a weaker border than the border between the calcified plaque and the soft plaque; (2) the other kind of approaches is to detect the calcified plaque directly, which always adopted a coarse-to-fine strategy to locate the calcification. In the first step, the classification algorithms, such as Otsu method [22], thresholding method based on 2-D Renyi's entropy [23] or k-means [24], were applied to the IVUS image s in order to coarsely specify the locations of calcified plaques. And in the second step, the acoustic shadowing would be the recognized by comparing the gray intensity of the calcified plaque with that of the acoustic shadowing [25], [26]. However, in the coarse detection, the ROI detection of the calcified plaque did not depend on its acoustic shadowing, and thus some bright non-calcified regions might be considered as the ROI of the calcification, such as collagen and guide-wire artifacts. Furthermore, the refinement relied on the image contrast between the calcified plaque and the acoustic shadowing, which is controlled by the adjustments of gamma curve in image acquisition [27], and thus the contrast may vary among different IVUS images. So the refinement of the calcified plaque should not rely on the gray intensity since it would reduce the performance of the algorithm.

In this paper, we develop a coarse-to-fine method to automatically detect the calcified plaque with acoustic shadowing in IVUS images in three steps: (1) the coarse detection of calcified plaque is implemented by Rayleigh mixture model (RMM) and Markov random field (MRF); (2) the refinement of calcified plaque is carried out by five predefined constraints; (3) the border of calcified plaque is detected by the graph searching algorithm. The validation of our method is performed over in-vivo IVUS images (DICOM format) acquired from eight patients. The results of our method are compared with the manual drawing results from one experienced cardiologist. In the experiments, the ability of our method to distinguish between the IVUS images with and without the calcified plaque is firstly evaluated by the sensitivity and specificity. Next, in the IVUS images with calcified plaque, two different evaluation methods, linear regression and Bland-Altman analysis, are performed in order to validate the correlation and the agreement between our method and the manual detection. In addition, we compute how much overlapping between two calcified plaques separately produced by our method and the manual detection, in order to evaluate the location accuracy of the calcified plaque detected by our method.

The rest of this paper is organized as follows: The second section discusses the proposed method, including the pixel classification by the RMM, the detection of the angular location of the calcified plaque, and the refinement and the border detection of the calcified plaque. The third section shows the experiments and results. The fourth section demonstrates the discussion, and the last section draws the conclusions of this study.

## Methodology

**Figure 1:**
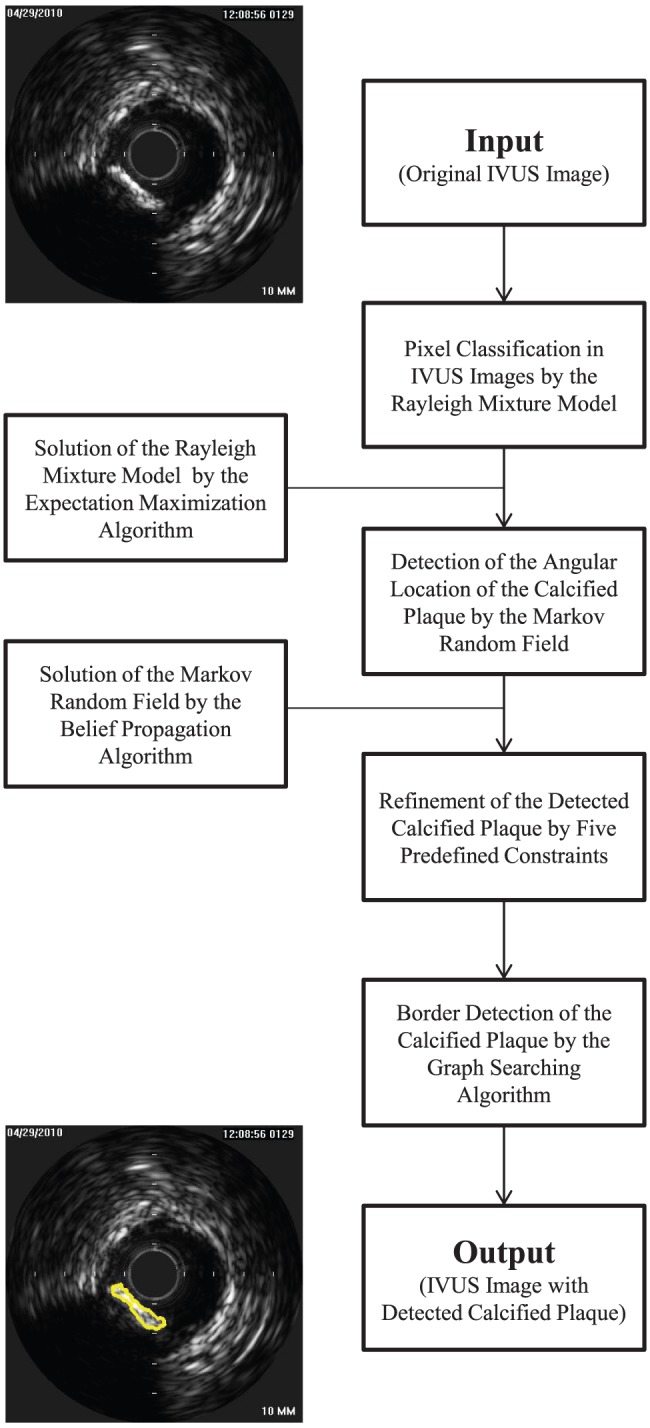
The flowchart of the proposed method.

In this section, we develop a method to detect the calcified plaque with acoustic shadowing in IVUS images. The flowchart of the proposed method is shown in Figure 1. First, a RMM is applied to cluster pixels in IVUS images in order to distinguish between the hyperechoic and hypoechoic regions, and solved by the EM algorithm. In the solution of the RMM, a new prior probability is produced based on the spatial relationship among neighboring pixels. Second, a MRF is employed to coarsely detect the location of the calcified plaque, and solved by the BP algorithm. In the solution of the MRF, the results of the RMM and a curve called maximum intensity curve (MIC) defined in the IVUS image are combined to compute the relationship between the observed variables and hidden variables, which can be considered as the prior information of the MRF. Third, the pseudo calcified plaques are removed by five predefined constraints. At last, the border of the calcified plaque is detected by the graph searching method algorithm [28] with cost function formatted by MIC and the image gradient.

### Pixel Classification in IVUS Images

Different vascular tissues appear in different gray levels and shapes in IVUS images, so we can use the RMM to describe the distribution of the image intensity and cluster pixels in IVUS images [29]. The RMM formulas are shown in Appendix A. There are three important issues in the RMM: (1) the selection of the number of the mixture components; (2) the construction of the prior probability; (3) the estimation of the parameters.

**Figure 2:**
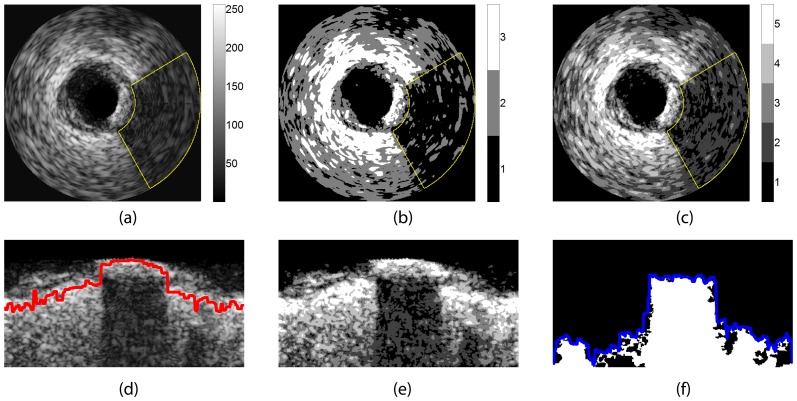
(a) The origin IVUS image. The region inside the yellow contour represents the acoustic shadowing behind the calcified plaque. (b) The image corresponding to (a) processed by the RMM with the class number 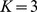, and the colorbar shows the colors of each class. The group including Class “1” contains 82.35% pixels inside the acoustic shadowing. (c) The image corresponding to (a) processed by the RMM with the class number 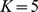, and the colorbar shows the colors of each class. The group including Class “1” and Class “2” contains 97.74% pixels inside the acoustic shadowing. (d) 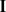. It corresponds to (a) in polar coordinates, and the red curve is the maximum intensity curve (MIC). (e) 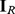. It corresponds to (c) in polar coordinates. (f) 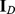. The white region is 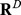, and the blue curve is 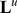.

First, the number of the mixture components 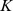 is empirically predefined as 5 in our study, different from 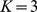 in previous approaches [30] [31], because the purpose of the pixel classification in the proposed method is to label pixels belonging to the acoustic shadowing into the same group, which can help the following steps for detecting the calcified plaque, rather than distinguish among three layers of the blood vessel [30], or among different types of atherosclerotic plaques [31]. Here we give a comparison of the RMM classification between 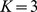 and 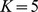, shown in Figure 2(b) and 2(c). For 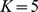, 97.74% of pixels in the acoustic shadowing are labeled into the same group, while for 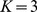, the percentage is only 82.35%. Therefore, the RMM with 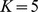 can more appropriately extract the acoustic shadowing in the IVUS image.

Second, different from many previous methods [30] [31], we add the neighboring relationship in the prior probability 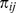, rather than assume the independence between pixels. The computation of 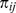 is shown in Appendix A.

Third, the parameters of the RMM are estimated by the maximum likelihood method (ML) [24], and the most direct way to solve the ML method is to compute the derivative of the likelihood function with respect to every unknown parameter. However, the computation is intractable due to the complexity of the class-conditional probability density 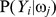 in Equation (8) and the prior 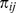 in Equation (13). Therefore, we apply the expectation-maximization algorithm (EM) [32] to solve the ML method. The EM algorithm is an iterative method for estimating the parameters of the maximum likelihood method. In each iteration, the EM algorithm can be divided into two step: E-step and M-step. The E-step is used to determine the objective function in this iteration, and the M-step is used to maximize the objective function in order to update the parameters. The details of the E-step and the M-step are shown in Appendix B.

From above, we present the detailed steps of the method for estimating RMM parameters in Appendix C. After the parameters of the RMM are determined, the pixels in the IVUS image can be clustered by the maximum a posterior estimation method (MAP) [24]: the *i*th pixel can be assigned to the *j*th class if
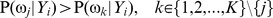
(1)


where
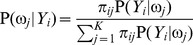
(2)


The results of the pixel classification by the RMM are shown in Figure 2(c).

### Detection of Angular Location of the Calcified Plaque

Because the growth of the calcified plaque is spatially continuous along the vessel wall, the pixels belonging to the same calcified plaque have the relationship along the angular direction in polar coordinates of the IVUS image. In order to model the relationship, we firstly transform the IVUS image and its corresponding image classified by the RMM both from the Cartesian domain to the polar domain. The IVUS polar image is denoted by 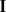 and its corresponding image after pixel classification is denoted by 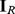, shown in Figure 2. Then every column of 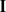 can be described by a random variable, and these random variables generate a MRF, which contains this kind of relationship [33]. Let the numbers of rows, columns and gray-scale value at the coordinate 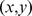 in 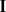 be 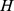, 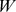 and 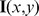, respectively, where 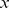 is the column coordinate and 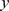 is the row coordinate. Then the hidden variables in the MRF are denoted by 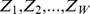, where 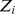 (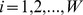) corresponds to the 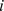
th column of 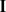. The domain of 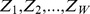 is -1 and +1, where 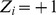 means the 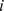
th column of 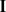 contains the calcified plaque, and 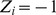 means it does not contain the calcified plaque. The neighborhood of 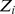 includes 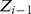 and 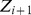. In addition, 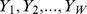 denote the observed variables corresponding to 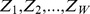, respectively. Through the MRF, the possibility of every column 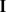 containing the calcified plaque with acoustic shadowing can be calculated. Therefore, the columns in 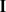 can be divided into two parts: one part contains the calcified plaque with acoustic shadowing and the other does not, where the columns including the calcified plaque can be considered as the angular location of the calcified plaque. There are two important issues in the MRF: the determination of the prior and the solution of the MRF.

1. The prior represents the relationship between the observed variables 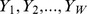 and the hidden variables 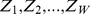. In our method, the prior 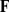 can be computed by the gray-scale information, the pixel classification by the RMM and the maximum intensity curve in 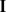, shown in Appendix D. The maximum intensity curve is defined in Definition 1.

**Definition 1** (Maximum intensity curve). *For 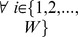, the pixel with the maximum gray-scale value in the 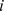
th column of* 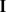 *can be found, and its row coordinate value is denoted by 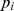. Let* 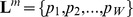, *and then* 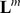 *is called the maximum intensity curve, abbreviated as MIC.*

2. The exact solution of the MRF is usually performed by the maximum likelihood (ML) or MAP parameter estimation [34]. In these methods, the parameter estimation can make the free energy of the MRF reach the extremum, and moreover it can be considered as a training process, because it requires the known observation (training data) to optimize the parameters. However, in many cases, there is no closed form solution of the MRF for the ML or MAP parameter estimation [34]. Thus, it is needed to use the approximate method to solve the MRF, and the training process aforementioned is implicit in the approximation process. The Bethe free energy is a common approximation to the free energy of the MRF [35], and has been proved to be equivalent to the belief propagation (BP) [36]. Therefore, we can use the BP algorithm to approximately solve the proposed MRF. The BP algorithm is an iterative “sum-product message passing” algorithm and applies a kind of variables called “belief” to approximate the probability of the random variables [37] [38]. The prior of the BP algorithm equals 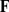 in Equation (28). The process of the proposed BP algorithm is shown in Appendix E.

The final beliefs can be denoted by 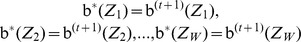 if the BP algorithm is converged in the 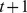 iteration. Then the belief of every column with the calcified plaque 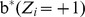 is binarized in order to find which columns of 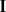 contain the calcified plaque, formulated as
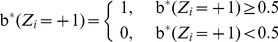
(3)


At last, we can find the angular location of the calcified plaque in 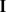 as follow: for two column 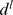 and 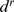 (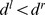), if 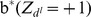, 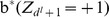,…, 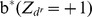 are all equal to 1, and 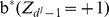, 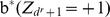 are both equal to 0, then the angular range between the 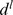
th column and the 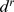
th column of 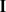 can be considered as the angular location of one calcified plaque, where 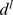 and 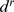 are called the left border and the right border of the calcified plaque, respectively.

### Refinement of the Calcified Plaque

The detection of the angular location of calcified plaques is affected by the sensitivity of MIC to the noise and the classification error from the RMM, which leads to the pseudo calcified plaques. Therefore, the refinement of the calcified plaque is needed. Here we define five constraints in order to refine the calcified plaque, that is, if a calcified plaque does not satisfy these constraints, then it can be considered as a pseudo calcified plaque. The details of the five constraints are shown in Appendix F. These constraints describe different aspects of the calcified plaque. Firstly, the Constraint 1 considers the brightness of the calcified plaque in IVUS images because the minimum threshold 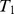 of pixel values limits the overall brightness of the calcified plaque. Then the Constraint 2 and the Constraint 3 aim to reduce the influence from other tissues on the recognition of the calcified plaque, because some tissues on adventitia, such as collagen, have high-level ultrasonic echo and thus can be represented as bright regions with high gray intensity, which may be mistaken as the calcified plaque. The Constraint 2, concerning the growth direction of the calcified plaque, limits the slope of two endpoints of the calcified plaques in order to recognize the bright region with too large slope in polar coordinate as the non-calcified region, because the calcified plaque grows along the vessel wall in histology. However, only relying on the Constraint 2, some non-calcified tissues on adventitia along the vessel wall still cannot be recognized. Therefore, the length of the calcified plaque must be considered. The limitation of the angular length of the calcified plaque along the polar axis in the Constraint 3 can exclude the bright regions with too long angular length. At last, the Constraint 4 and the Constraint 5 focus on the acoustic shadowing behind the calcified plaque. Because the calcified plaque can reflect most ultrasonic signals, the acoustic shadowing behind the calcified plaque is darker than its nearby tissues. Therefore, in the Constraint 4, the limitation of the minimum difference between the brightness of the acoustic shadowing and the nearby tissues can facilitate to recognize the shadowed regions on adventitia, ensuring that every detected calcified plaque has acoustic shadowing behind it. The Constraint 5 aims to recognize the pseudo acoustic shadowing through the shape of the acoustic shadowing, where Equation (47) represents the closeness between the calcified plaque and its acoustic shadowing because the amplitude of the ultrasonic signal behind the calcified plaque decreases sharply. Moreover, the ultrasound wave is omnidirectionally emitted from the catheter, and thus the acoustic shadowing is rectangle-like in polar coordinates. Equation (48) is used to describe the similarity between the acoustic shadowing and the rectangle-like shape in polar coordinates.

### Border Detection of the Calcified Plaque

**Figure 3:**
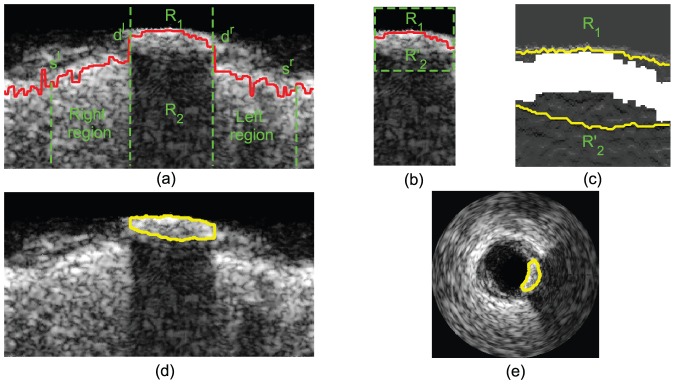
(a) The red curve is MIC. 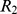
 contains the acoustic shadowing and part of the calcified plaque. The column coordinates of four vertical dashed lines from left to right are 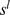, 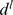, 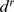, 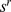, respectively. (b) The subregion composed of 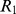 and 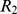 extracted from (a). And the region between the the green dashed rectangle and the red curve (MIC) is 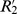. (c) The cost function of 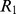 and 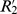, and their gap is the MIC in (b). The yellow curves are the optimal paths acquired by the graph searching algorithm in 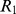 and 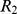, respectively. (d) The yellow curve is the detected calcified plaque in polar coordinate. (e) The yellow curve is the detected calcified plaque in Cartesian coordinate.

After the refinement, we detect the border of the calcified plaque, which can be divided into four part: the left border, the right border, the upper border and the lower border. The left border 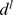 and the right border 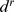 represent the angular location of the calcified plaque, and have been computed in the above. Therefore, only the upper border and the lower border of the calcified plaque should be detected in this part. In order to trace the two borders, we employ the graph searching algorithm [28], which is a searching algorithm to find the optimal path in the weighted graph (cost function) connecting two endpoints. Because MIC crosses through the calcified plaque, we consider the upper border is inside a region, denoted by 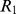, above MIC and between the 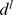
th column and the 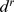
th column, and consider the lower border is inside a region, denoted by 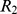, below MIC and between the 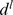
th column and the 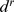
th column. 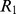 and 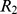 are shown in Figure 3(a). Then the graph searching algorithm is separately applied to 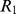 and 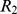 to extract the upper border and the lower border of the calcified plaque. There are two important issues in the graph searching algorithm: the selection of the two endpoints and the formulation of the cost function.

(1) In order to generate a traced border, two endpoints of the border, called the beginning point and the ending point, should be acquired in advance for the graph searching algorithm. With respect to 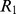 or 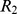, the column coordinates of the two endpoints are 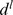 and 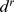. In addition, the upper border and the lower border of the calcified plaque are the junctions between the calcified plaque and non-calcified tissues, which may be influenced by the noise and tissues with high level echo. Therefore, in order to reduce above influence, the row coordinates of the endpoints are not specified beforehand, that implies the optimal border traced by the graph searching algorithm has the global minimum cost.

(2) Because the graph searching algorithm is separately applied to 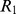 and 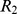, two cost functions should be computed applied to 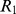 and 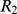, respectively. With respect to 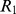, the upper border can be considered as the transition between the bright region and the dark region in the IVUS image. Therefore, the cost function in 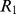 can be constructed based on the gradient information, formulated as
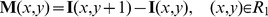
(4)


With respect to 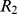, the acoustic shadowing occupies most of its area, and moreover, the region of acoustic shadowing far from the catheter cannot provide useful information for the detection of the lower border; on the contrary, the noise in the region may affect the tracing of the lower border. Therefore, we extract a subregion 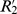 from 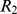 for tracing the lower border of the calcified plaque in order to reduce the influence from the noise. 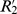 is defined as the region between the 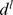
th column and the 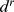
th column, and between MIC and the 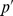
th row computed in Equation (46). 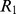, 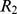 and 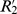 acquired from 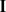 are shown in Figure 3(a) and Figure 3(b). Because the lower border can also be considered as a dark-bright transition, the cost function in 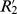 can be defined similarly to Equation (4) as
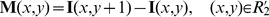
(5)


The cost function 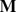 in Figure 3(b) is shown in Figure 3(c).

The results of the graph searching algorithm is shown in Figure 3(d), where the calcified plaque is the region inside the yellow contour. In order to reconstruct the border of calcified plaque in original IVUS images, it is transformed from polar domain to Cartesian domain, shown as the yellow contour in Figure 3(e).

## Results

The data acquisition was performed with the approval of the Health Science Research Ethics Committee of the Department of Cardiology at Guangdong General Hospital, and the participants provided written informed consent before beginning the acquisition. All the IVUS data were collected over a Volcano machine (Volcano Corp., Rancho Cordova USA) with a pullback speed of 0.5mm/s using a 20MHz solid-state IVUS catheter. The IVUS images were reconstructed and processed at a commercially available IVUS console (In-Vision Gold, Volcano Corp., Rancho Cordova USA) and saved as DICOM format into CDs for off-line analysis. All the codes in this study were implemented by Matlab R2012a on a desktop computer with Intel(R) Xeon(R) CPU E5-2650(2.00 GHz) and 32GB memory. In our experiments, calcified plaques detected by our method were compared with those manually detected by one cardiologist. Totally 996 in-vivo IVUS images are used in our study, where 498 images contain the calcified plaque with acoustic shadowing (considered as the experimental group), and other 498 images do not contain any calcified plaque with acoustic shadowing (considered as the control group), and there are three kinds of imaging diameter (8mm, 10mm and 12mm) in these IVUS images. The selection protocol of the IVUS images contains: (1) every calcified plaque in IVUS images should have the acoustic shadowing behind it; (2) all IVUS images are collected at different times from eight patients.

**Figure 4:**
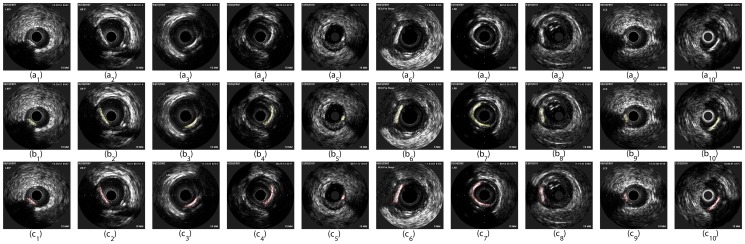
Some results of the detection of the calcified plaque. (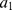)-(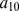) are original IVUS images. (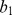)-(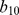) are the results of our method corresponding to (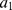)-(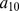), respectively, where the region within the yellow contour is the calcified plaque. (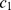)-(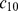) are the results of the manual drawing method by a cardiologist corresponding to (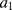)-(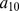), respectively, where the region within the red contour is the calcified plaque.

**Figure 5:**
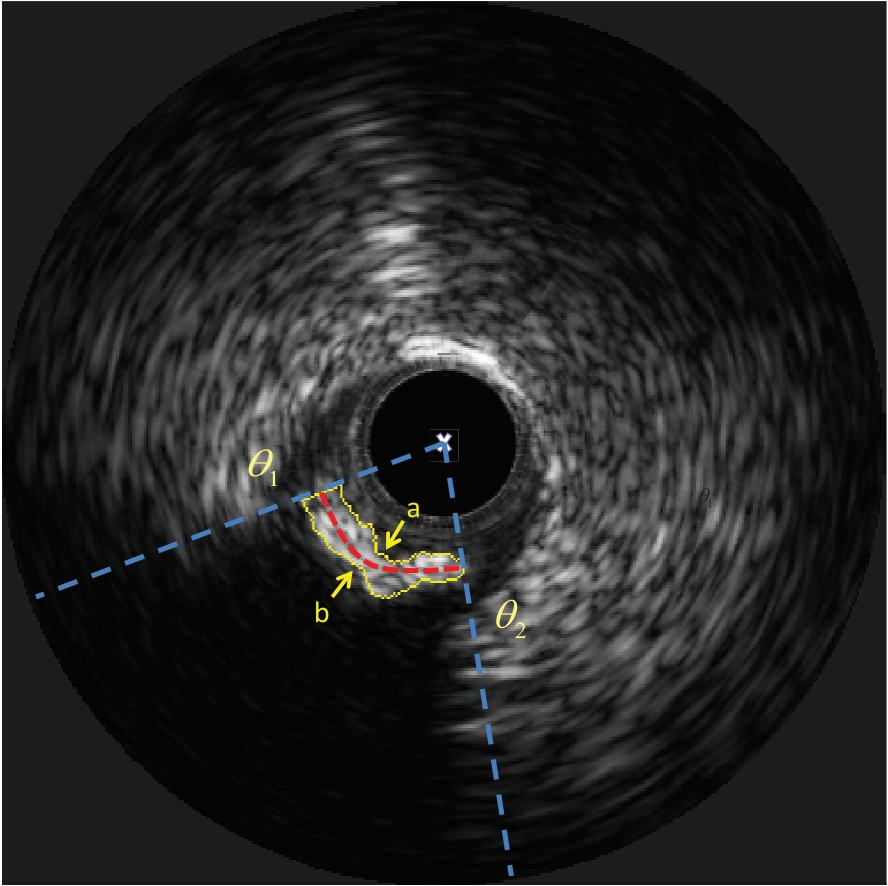
The white “×” represents the catheter center. The region within the yellow closed curve represents the detected calcified plaque. The yellow curve “a” and “b” are the leading edge and the trailing edge of the calcified plaque. The region between 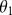 and 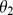 (two blue dashed lines) represents the angular location of the plaque. AL is angular length of the red dash line. AC is the mean of 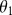 and 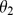. PA is the area within the yellow closed curve. PT is the mean distance between the curve “a” and the curve “b”. DC is the mean distance between the curve “a” and the catheter center.

The results of detecting the calcified plaque in some IVUS images are shown in Figure 4. The performance of our method was evaluated in two aspects: one was to evaluate the ability of our method to correctly distinguish between IVUS images with and without the calcified plaque; the other was to evaluate the location accuracy of the calcified plaque. Firstly, the ability to correctly identify IVUS images with the calcified plaque or without was evaluated by the sensitivity and specificity. The sensitivity represents the percentage of correctly identified IVUS images with the calcified plaque in the experimental group, and the specificity represents the percentage of correctly identified IVUS images without the calcified plaque in the control group, which can be computed by true positive (TP), false positive (FP), true negative (TN) and false negative (FN):
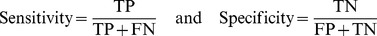
(6)where TP is the number of IVUS images with the calcified plaque correctly identified, TN is the number of IVUS images without the calcified plaque incorrectly identified, FP is the number of IVUS images with the calcified plaque incorrectly identified, and FN is the number of IVUS images without the calcified plaque correctly identified. Table 1 shows the sensitivity and the specificity tested over all patients are 94.68% and 95.82%, respectively. Secondly, the location accuracy of correctly identified calcified plaques was quantified by five measurements: plaque area (PA), angular length (AL), angular center (AC), plaque thickness (PT) and the distance to catheter (DC), shown in Figure 5. PA can measure the size of the calcified plaque. AL and AC can measure the angular location (the location along the angular direction) of the calcified plaque between two angles 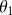 and 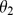, formulated as
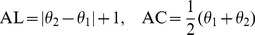
(7)


**Table 1:** The results of distinguishing between the IVUS images with and without the calcified plaque.

Sensitivity	Specificity
94.68%	95.82%

The sensitivity represents the accuracy rate of identifying the IVUS images with the calcified plaque, and the specificity represents the accuracy rate of identifying the IVUS images without the calcified plaque.

**Figure 6:**
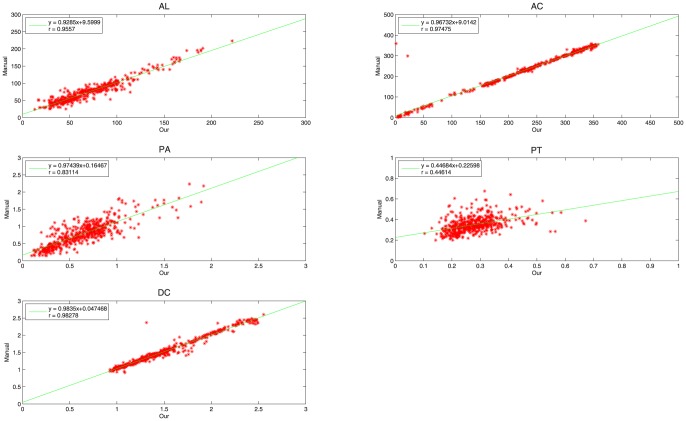
The results of the linear regression with respect to AL, AC, PA, PT and DC for all patients. The horizontal axis and vertical axis represent results acquired from our method and the manual drawing, respectively.

**Figure 7:**
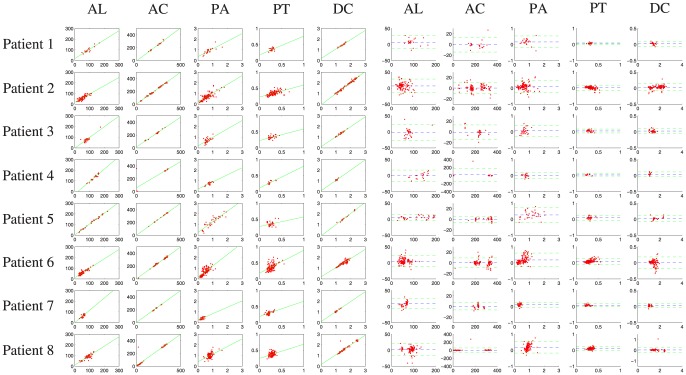
The between-patient results of our method separately from Patient 1 to Patient 8 with respect to AL, AC, PA, PT and DC. The left five columns represents the results of the linear regression. The right five column represents the results of the Bland-Altman analysis, where the blue dashed lines and the green dashed lines indicate the location of MEAN and 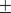
2
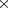
STD, respectively.

**Figure 8:**
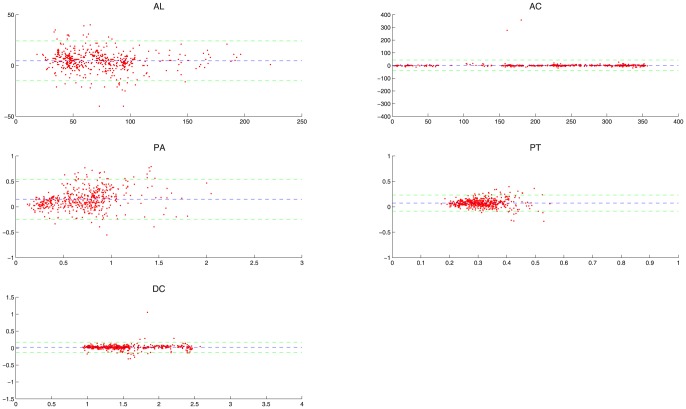
The Bland-Altman plot with respect to AL, AC, PA, PT and DC for all patients. The blue dashed lined and the green dashed lines indicate the location of MEAN and 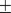
2
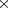
STD, respectively.

PT and DC can measure the radial location (the location along the radial direction) of the calcified plaque, where DC equals the distance between the leading edge of the calcified plaque and the catheter's center. These measurements calculated from our method and the manual drawing method were compared separately by two different evaluation methods: linear regression and Bland-Altman analysis [39], in order to analyze their correlation and agreement, respectively. In every evaluation method, we firstly analyze the comparative results on all IVUS images in order to evaluate the overall performance of our method, and then on IVUS images from every patient in order to evaluate the between-patient difference of our method. In linear regression, the correlation coefficient 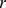 and the root-mean-square error (RMSE) are used to investigate the overall correlation between our method and the manual drawing method. Figure 6 shows the overall correlation. For AL, AC, PA, PT and DC, the values of 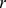 are 0.9557, 0.9747, 0.8311, 0.4461 and 0.9828, respectively, and the values of RMSE are 9.5055, 20.5260, 0.1964, 0.0674 and 0.0758, respectively. Figure 7 shows the between-patient difference for the five measurements. The standard deviation of 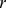 with respect to the five measurements is smaller than 0.15. Table 2 shows the corresponding numerical results. In the Bland-Altman analysis, four indices, 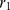, 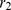, 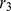 and CI, are used to investigate the agreement between our method and the manual drawing method. Let 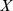 be the difference between same two measurements computed separately by our method and by manual, and let 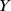 be the mean value of the two measurements. CI represents the 95% confidence interval of 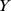, formulated as 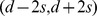, where 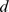 and 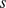 are the mean value and standard deviation of 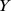, respectively. 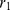 represents the ratio of d to the mean value of 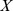. 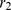 represents the ratio of the maximum absolute value of 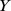 to the mean value of 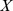. 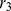 represents the frequency of the points without CI in the Bland-Altman plot. Figure 8 shows the overall agreement between our method and the manual drawing method. With respect to the five measurements, 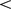
7.1% of results fall without CI in the Student t-test. Figure 7 shows the between-patient difference of our method. The standard deviation of the number of results within the 95% confidence interval with respective to the five measurements is smaller than 2.61%. Table 3 presents the corresponding numerical results. Additionally, the overlap between two calcified plaques detected by our method and drawn by manual separately can also represent the location accuracy of the calcified plaque, which is measured by the sensitivity and specificity. The sensitivity and specificity can also be calculated by Equation (6), where TP is the number of pixels inside the calcified plaque correctly identified, FP is the number of pixels inside the calcified plaque incorrectly identified, FN is the number of pixels outside the calcified plaque incorrectly identified, and TP is the number of pixels outside the calcified plaque correctly identified. Table 4 shows that the mean values of the sensitivity and specificity in the measurement of the overlap are 83.79% and 99.74%, respectively.

**Table 2:** The results of the linear regression.

Patient	Measure	Parameter		
		Linear function ( 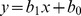 )	r	RMSE
1	AL	y = 0.8683x+18.8034	0.9176	9.5360
	AC	y = 1.0302x−6.6298	0.9930	6.5603
	PA	y = 0.7199x+0.3686	0.7778	0.1643
	PT	y = 0.5830x+0.1788	0.4899	0.0374
	DC	y = 0.8776x+0.1959	0.9688	0.0329
2	AL	y = 0.7749x+17.8654	0.8498	9.6752
	AC	y = 0.9927x+1.7758	0.9977	4.8579
	PA	y = 0.8296x+0.2014	0.8040	0.1839
	PT	y = 0.3747x+0.2250	0.5692	0.0509
	DC	y = 1.0427x−0.0476	0.9934	0.0517
3	AL	y = 0.9992x−2.1011	0.8546	12.5169
	AC	y = 0.9278x+15.1089	0.9939	5.5919
	PA	y = 0.7936x+0.1924	0.5879	0.1754
	PT	y = 0.3611x+0.2308	0.4738	0.0438
	DC	y = 0.9338x+0.1132	0.9784	0.0364
4	AL	y = 1.1654x−22.9853	0.9648	7.8523
	AC	y = 0.8161x+59.2887	0.8583	75.2922
	PA	y = 0.6633x+0.2424	0.7808	0.0776
	PT	y = 0.6770x+0.1162	0.7378	0.0307
	DC	y = 0.9656x+0.0807	0.8524	0.0333
5	AL	y = 1.0189x+3.3029	0.9965	7.8523
	AC	y = 0.9977x+0.4821	0.9992	75.2922
	PA	y = 0.9966x+0.2681	0.8982	0.0776
	PT	y = 0.2963x+0.2759	0.2631	0.0307
	DC	y = 1.0439x−0.0468	0.9942	0.0333
6	AL	y = 0.8803x+11.6050	0.8962	8.1192
	AC	y = 0.9931x+1.8185	0.9973	4.6016
	PA	y = 1.0713x+0.1476	0.7338	0.2136
	PT	y = 0.7095x+0.1835	0.4835	0.0907
	DC	y = 0.8533x+0.2055	0.9325	0.0831
7	AL	y = 1.1503x+0.0611	0.8258	7.5915
	AC	y = 0.9918x+2.4934	0.9897	3.4321
	PA	y = 0.6027x+0.2296	0.5284	0.0704
	PT	y = 0.4754x+0.1926	0.5728	0.0350
	DC	y = 0.9345x+0.0977	0.9589	0.0263
8	AL	y = 0.8802x+11.8663	0.9057	8.8089
	AC	y = 0.9817x+7.5092	0.9809	26.1497
	PA	y = 0.7093x+0.4292	0.5125	0.1789
	PT	y = 0.4396x+0.2494	0.3378	0.0542
	DC	y = 0.9180x+0.1622	0.9631	0.1034
1–8	AL	y = 0.9285x+9.5999	0.9557	9.5055
	AC	y = 0.9673X+9.0142	0.9747	20.5260
	PA	y = 0.9744x+0.1647	0.8311	0.1964
	PT	y = 0.4468x+0.2260	0.4461	0.0674
	DC	y = 0.9835x+0.0475	0.9828	0.0758

**Table 3:** The results of the Bland-Altman analysis.

Patient	Measurement	Index			
		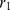	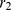	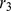	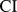
1	AL	0.0787	0.2401	8.33%	[−13.4334,27.8500]
	AC	0.0015	0.0527	4.17%	[−14.1180,13.4930]
	PA	0.1972	0.4511	8.33%	[−0.2110,0.5342]
	PT	0.2199	0.4186	8.33%	[−0.0158,0.1489]
	DC	0.0176	0.0528	8.33%	[−0.0519,0.1014]
2	AL	0.1367	0.5429	7.63%	[−14.4069,28.5086]
	AC	0.0014	0.0443	7.63%	[−9.5267,10.1030]
	PA	0.1505	0.6993	5.93%	[−0.2849,0.4817]
	PT	0.0594	0.5059	4.24%	[−0.1362,0.1763]
	DC	0.0163	0.0761	6.78%	[−0.0804,0.1399]
3	AL	0.0271	0.2755	3.33%	[−27.6284,23.2950]
	AC	0.0041	0.0452	10.00%	[−14.7981,12.9648]
	PA	0.0871	0.5647	3.33%	[−0.3048,0.4221]
	PT	0.1773	0.4228	6.67%	[−0.0691,0.1768]
	DC	0.0185	0.0569	6.67%	[−0.0532,0.1030]
4	AL	0.0080	0.1287	0.00%	[−19.2427,17.1374]
	AC	0.0681	0.0497	5.26%	[−148.9124,181.7545]
	PA	0.0160	0.2434	5.26%	[−0.2010,0.1769]
	PT	0.1532	0.4028	5.26%	[−0.0316,0.1105]
	DC	0.0411	0.0876	5.26%	[−0.0242,0.1129]
5	AL	0.0470	0.1296	0.00%	[−4.5308,15.4132]
	AC	0.0006	0.0192	2.94%	[−5.6463,5.2640]
	PA	0.2400	0.6115	5.88%	[−0.1914,0.7210]
	PT	0.3187	0.8188	5.88%	[−0.0561,0.2493]
	DC	0.0146	0.0478	8.82%	[−0.0584,0.1055]
6	AL	0.0857	0.3624	6.03%	[−11.9474,21.8785]
	AC	0.0001	0.0300	10.34%	[−9.3096,9.2579]
	PA	0.3247	1.0545	6.03%	[−0.2494,0.6110]
	PT	0.3313	0.8845	5.17%	[−0.0809,0.2928]
	DC	0.0024	0.1199	6.03%	[−0.1793,0.1859]
7	AL	0.1407	0.3534	6.67%	[−6.8775,24.3886]
	AC	0.0028	0.0331	4.44%	[−6.3087,7.5976]
	PA	0.3194	0.6291	6.67%	[−0.0417,0.2662]
	PT	0.3116	0.6289	4.44%	[−0.0098,0.1689]
	DC	0.0286	0.0752	2.22%	[−0.0248,0.0847]
8	AL	0.0212	0.2238	7.14%	[−16.6264,20.2335]
	AC	0.0165	0.0716	0.89%	[−49.1859,56.3466]
	PA	0.2173	0.6135	3.57%	[−0.1742,0.5658]
	PT	0.2860	0.6050	3.57%	[−0.0268,0.2129]
	DC	0.0160	0.0856	0.89%	[−0.1916,0.2448]
1–8	AL	0.0630	0.3030	7.03%	[−15.0437,24.1883]
	AC	0.0063	0.1166	0.40%	[−40.0689,43.0067]
	PA	0.2113	0.7590	6.02%	[−0.2448,0.5420]
	PT	0.2295	0.7184	5.82%	[−0.0864,0.2307]
	DC	0.0148	0.0911	4.02%	[−0.1299,0.1749]

**Table 4:** The results of the overlapping rate between the calcified plaques detected by our method and drawn by manual.

Patient	Sensitivity	Specificity
1	85.60%±7.23%	99.63%±0.18%
2	84.63%±12.05%	99.79%±0.15%
3	76.47%±12.96%	99.80%±0.13%
4	78.95%±8.00%	99.83%±0.08%
5	85.14%±11.44%	99.60%±0.20%
6	84.10%±10.61%	99.74%±0.19%
7	88.03%±7.18%	99.85%±0.07%
8	85.65%±8.70%	99.68%±0.17%
1–8	83.79%±12.61%	99.74%±0.18%

The sensitivity represents the percentage of the pixels correctly identified inside the calcified plaque, and the specificity represents the percentage of the pixels correctly identified outside the calcified plaque.

In addition, Table 5 compares the computational costs of our method separately applied to the experimental group and the control group. In the experimental group, the mean value and standard deviation of the computational cost are 6.81s and 0.21s, respectively. And in the control group, the mean value and standard deviation are 5.26s and 0.08s, respectively.

**Table 5:** The computing time of the proposed method is formulated as mean 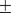 standard deviation measured by the second.

	RMM	MRF	Refinement	Border Detection	Other	Total
Experimental Group	2.43 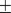 0.14	3.35 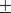 0.02	0.03 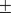 0.01	0.15 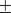 0.01	0.91 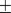 0.01	6.81 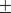 0.21
Control Group	2.05 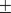 0.05	2.81 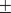 0.04	0.02 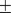 0.01	0.0009 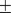 0.0001	0.49 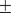 0.01	5.26 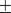 0.08

“RMM”,“MRF”,“Refinement”,“Border Detection” represent the computing time in the pixel classification by the RMM, the angular location detection of the calcified plaque by the MRF, the refinement of the calcified plaque and the border detection by the graph searching method, respectively. “Total” represents the computing time of the total method, and “Other” represents the computing time of the total method excluding the above parts.

## Discussion

In this section, the accuracy and robustness of our method, the computational cost, the limitation and the future work will be discussed.

### Accuracy

The accuracy of our method is evaluated through comparing the results from the manual drawing by one cardiologist and from the computer-aided automatic detection by our method. The evaluation of accuracy can be divided into two parts: one is to test the accuracy of distinguishing between IVUS images with and without the calcified plaque; the other is to evaluate the location accuracy of the calcified plaque drawn by manual and detected by our method. Firstly, the identification of the IVUS image with or without the calcified plaque can be considered as a binary classification, and thus the sensitivity can be used to represent the correct classification rate of the images with the calcified plaque and the specificity can be used to represent the correct classification rates of the images without the calcified plaque. In Table 1, the sensitivity and specificity computed from all IVUS images are 94.68% and 95.82%, respectively, representing that our method has good ability to distinguish between the normal vascular cross section and the pathological vascular cross section with respect to the calcification. Secondly, the location accuracy of the calcified plaque is evaluated separately by linear regression and Bland-Altman analysis. The linear regression focuses on the correlation between our results and manual drawing results by the correlation coefficient 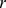 and RMSE. In Table 2, we can see that our results and the manual drawing results have high statistical relationship in detecting the angular location of the calcified plaque because 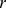 equals 0.9557 for AL and equals 0.9747 for AC. And our method also has high correlation with the manual drawing method in detecting radial location of the calcified plaque because 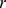 equals 0.9828 for the distance DC from its leading edge to the catheter (shown in Figure 5). However, the correlation for PT has low level (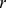 = 0.4461), implying that the detection of the trailing edge of the calcified plaque do not have enough accuracy (shown in Figure 5). That is because the ultrasonic signals transmitted from the catheter are almost completely blocked by the calcified plaque, forming a shadow behind the calcified plaque that makes it difficult to estimate the real thickness of the calcium. Moreover, the high correlation for PA (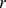 = 0.8311) represents the sizes of the same calcified plaque detected by manual and our method are close to each other. In addition, the values of 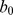 are all larger than zero for the five measurements, which means that the five measurements computed by our method is the underestimation of the manual drawing results. In the Bland-Altman analysis, we evaluate the agreement between our method and the manual drawing method. The values of 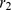 for AL, PA, PT are 0.3030, 0.7590, 0.7184, respectively, representing that the maximum difference between our method and manual drawing method is not small, and however the values of 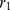 for the three measurements (0.0630, 0.2113, 0.2295) show that the variation in measuring AL, PA and PT are small, implying that 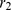 for AL, PA, PT are large only in a small amount of IVUS images. Moreover, 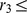
7.03% for AL, AC, PA, PT and DC in the Bland-Altman analysis shows most of our results fall into 95% confidence interval with high reliability in the Student t-test. In addition, the overlapping rate between the calcium regions detected separately by manual and by our method can be measured by the sensitivity and specificity, which can represent how many image pixels belong to the calcified plaque. In Table 4, the mean values of the sensitivity and the specificity are respectively 83.79% and 99.74%, representing that the difference between the calcification regions obtained by manual and by our method is at a low level, and furthermore the number of correctly identified pixels inside the calcium region is smaller than the number of correctly identified pixels outside the calcium region, implying that the calcified plaque detected by our method is a little underestimation of the plaque manually drawn. In conclusion, we can consider that the accuracy of the detection of the calcified plaque by our method is at a high level.

### Robustness

With respect to the robustness of our method, it is investigated from two aspects: the process of our method and the between-patient performance of our method. Firstly, the kernel of our method includes the pixel classification by the RMM, the angular location detection by the MRF, and the refinement of the calcified plaque by the predefined constraints. Due to the superposition of ultrasonic signals in the vessel and the influence from the speckle noise, the pixel intensity of different tissues in the IVUS image can be described by the probabilistic model. Comparing with the widely used Gaussian mixture model [40] [41], the RMM is more appropriate to describe the distribution of pixel intensity in the IVUS image because the statistics of the pixel intensity relies on the speckle induced by a lot of randomly located scatters, and can be well modeled by the Rayleigh distribution. The pixel classification by the RMM facilitates to the subsequent step of the proposed method for the location of the calcified plaque, because it can make the class number of pixels decrease from the level of the gray intensity to the component number of RMM, which can also be considered to improve the between-class difference and reduce the within-class difference of the image pxiels. Moreover, unlike the RMM applied in IVUS previously [9] [10] [42], we add the spatial relationship into the RMM classification. Because the vascular tissues have the spatial continuity in anatomy, the pixel values of the neighboring pixels in IVUS images are statistically dependent. The dependence can help to improve the robustness of the RMM classification to the noise because the prior probability generated by the dependent relationship in Equation (10) - Equation (13) has an effect on the local smooth. Similarly, the calcified plaque can also be considered to have the angular spatial relationship because it grows along the vessel wall. Therefore, we consider that the calcified plaque has the Markovianity along the angular axis of the IVUS image in polar coordinate, and the MRF can be used to determine the angular location of the calcified plaque based on the results of the RMM classification. However, the angular location of the calcified plaque is still influenced by the noise and error in the RMM classification. Therefore, the refinement of the calcified plaque is needed. In the refinement, five predefined constraints are proposed by considering different aspects of the calcified plaque: the gray intensity, the growth direction, the angular length and the corresponding acoustic shadowing. And any calcified plaque not satisfying these constraints can be considered as the pseudo calcification and then removed. In addition, different some previous works [13], our detection method does not rely on the beforehand detection of the lumen border and/or the media-adventitia border, which can save the computation cost and moreover avoid the error in the detection of the calcified plaque resulting from the error of the vessel border detection.

Secondly, the between-patient difference of our method is also investigated to further examine the robustness of our method. In the linear regression and the Bland-Altman analysis, the performances of our method tested separately from Patient 1 to Patient 8 are compared. Table 2 presents the standard deviation of the correlation coefficient 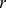 in linear regression for every measure (AL, AC, PA, PT and DC) in different patients are smaller than 0.15. Table 3 shows the standard deviation of 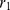, 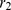 and 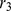 in the Bland-Altman analysis for every measure in different patients are smaller than 0.11, 0.23, and 2.61%, respectively, which means that there is no significant between-patient difference of our method with respect to the correlation and the agreement. In addition, the overlap between the calcified plaques detected by our method and by manual drawing in different patients is shown in Table 4, where the standard deviation of the mean values of the sensitivity and specificity (3.85% and 0.09%) represents that overlapping rate has small difference in different patients. Therefore, we can conclude that our method has similar performance in different patients.

### Computational Cost

The computational cost of our method is presented in Table 5. Firstly, the computation cost over the experimental group is compared with the control group. The result shows that our method runs faster about 1.6s in the control group than in the experimental group, because the refinement of the calcified plaque will spend less time and the border tracing by the graph searching will not be executed when the IVUS image does not contain the calcified plaque with acoustic shadowing. Secondly, by comparing different parts in our method, we can see that the pixel classification by the RMM and angular location detection of the calcified plaque by MRF take the two most computational costs in the process of our method (35% and 49% in the experimental group and 39% and 53% in the control group), because the two parts of our method contain plenty of iteration operations. Moreover, the standard deviations of the cost in each parts of our method are small (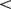
0.14s), and that implies the computational cost is hardly affected by the variation of the plaque size. In addition, the cost of our method (“others” in Table 5) excluding the RMM classification, the angular location detection by MRF, the refinement and the border detection spends 13% and 9% of the total cost in the experimental group and the control group, respectively, and it might be reduced by optimizing the programming code in order to improve the computational efficiency.

### Future Work

In the future, we will improve the efficiency of our method in two aspects: one is to improve the model for IVUS; the other is to improve the prior knowledge about the plaque. Firstly, we will try to apply different probabilistic mixture models for more appropriately describing the pixel intensity in the IVUS images, and use more complex graphical models to instead of the MRF in order to improve the detection accuracy of the calcified plaque. Secondly, we will add some high-level prior knowledge into the RMM in order to consider each kind of tissues as a whole for facilitating the pixel classification. In addition, in order to combine the above two aspects, the supervised scheme can be used by training the plaque models based on the more complex predefined features of the calcified plaque for better performance of our method.

## Conclusion

The computer-aided detection of the calcified plaque with acoustic shadowing is vital in the quantitative analysis of IVUS images. In this paper, a method based on the Rayleigh mixture model, the Markov random field and the graph searching algorithm is proposed for the automatic detection of the calcified plaque. Additionally, the proposed method includes a refinement strategy to remove pseudo calcified plaques based on the predefined constraints about the calcification. The performance evaluation between the proposed automatic detection method and the manual drawing method (by one cardiologist) was performed over 996 in-vivo coronary IVUS images acquired from 8 patients with two aspects. The first aspect is the evaluation of the accuracy to correctly identify the IVUS images with or without the calcified plaque, and it was measured by the test of the sensitivity and specificity. The results shown that there is high correct rate of identifying the IVUS image including or excluding the calcified plaque with sensitivity 94.68% and specificity 95.82%. The other aspect is the evaluation of the accuracy to locate the calcified plaque by the linear regression, the Bland-Altman analysis and the overlapping rate between the calcified plaques separately detected by our method and by manual drawing. The linear regression shown that the purposed automatic method is correlated very well with the manual drawing method over four measurements (angular length, angular center, plaque area and the distance to catheter) with high correlation coefficients, and the Bland-Altman analysis demonstrated a high level of agreement between our method and the manual drawing method with 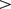
93% results falling within the 95% confidence interval. In addition, the sensitivity (83.79%) and specificity (99.74%) used to measure the overlapping rate shown that the calcified plaques detected by our method and drawn by manual measured are matched well. These results demonstrated the effectiveness of our method in the detection of the calcified plaque with acoustic shadowing in IVUS images.

## Appendices

### A Constructing the RMM model

The RMM can be formulated as,
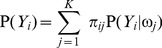
(8)where 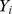 is the 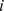
th pixel in the IVUS image, 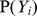 is the probability density function of 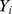 in the RMM, 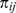 is the prior probability, 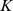 is number of mixture components in the RMM, 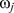 is the parameter vector of the *j*th component, and 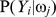 is the probability density function of 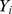 in the *j*th component. 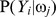 can be formulated by the Rayleigh distribution with parameter 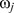 [43],
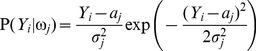
(9)where 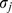 is the mode, 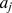 is the translation component and 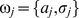.

The construction of the prior 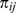 in the RMM considers the neighboring relationship among pixels in IVUS images, inspired by [44] and computed as follows:

1. Define the weight of the 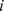
th pixel in the 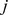
th component of the RMM as 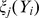,
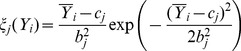
(10)where 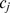 and 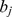 (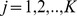) are parameters, and 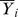 is the mean value of pixels in the neighborhood 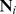 of the 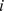
th pixel, formulated as
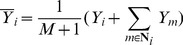
(11)where 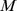 is the number of pixels in the neighborhood 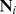.

2. Define the weight of the 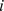
th pixel and its neighborhood 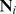 in the 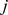
th component of the RMM as 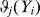,
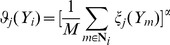
(12)where 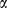 is used to control the function shape of 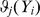.

3. Define the prior probability 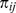 as
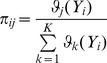
(13)


In Equation (13), the normalization of 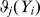 aims to translate the range of 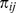 into 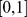.

### B Formulating the E-step and the M-step in the EM algorithm

In the E-step, the objective function of the EM algorithm for the RMM can be formulated as
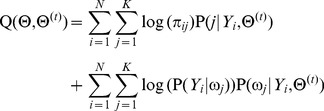
(14)where 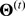 is the known parameters in the *t*th iteration, 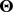 is the unknown or updating parameters in the *t*th iteration, 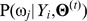 is the posterior distribution and 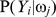 is class-conditional probability density. Because the first term in the righthand of Equation (14) does not contain the parameters 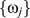, and the second term of that does not contain the prior probabilities 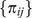, the maximization of the objective function 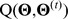 can be simplified to separately maximize the first term in the righthand of Equation (14) with respect to the parameter set 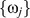, and to maximize the second term of that with respect to the prior 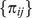. In the M-step, we employ the steepest-descent method [45] to maximize the objective function 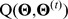 in order to update the parameter set 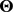,
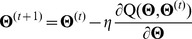
(15)where 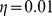 is the learning rate. Let 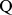 be the abbreviation of 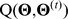, and 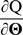 can be written as a matrix,
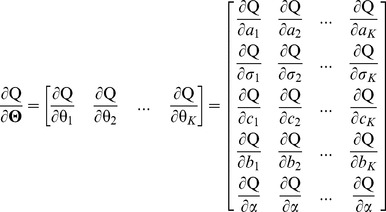
(16)



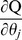
 in Equation (16) can be calculated as follows:

1. The partial derivative of 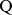 with respect to 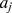 is
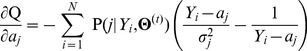
(17)


2. The partial derivative of 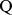 with respect to 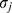 is
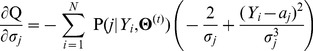
(18)


3. The partial derivative of 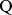 with respect to 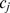 is
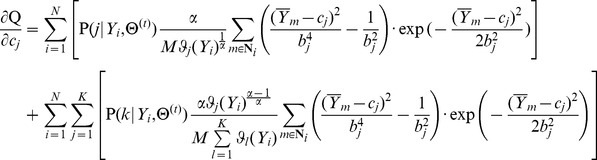
(19)


4. The partial derivative of 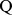 with respect to 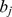 is
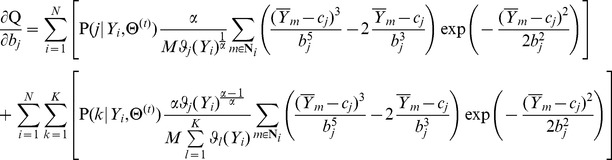
(20)


5. The partial derivative of 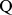 with respect to 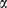 is
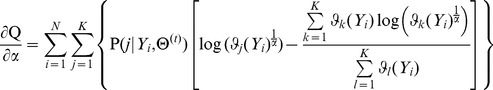
(21)


### C Estimating the RMM parameters by the EM algorithm

The estimation of the RMM parameters by the EM algorithm can be divided into the following steps:

Step 1: initialize the parameter set 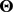.

(1) The class number 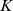 is set at 5 and 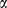 is initialized as 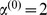. Then the k-means algorithm [24] is used to cluster pixels in the IVUS image based on the gray intensity in order to find the mean gray-scale values of 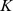 classes 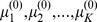, and denote 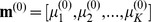.

(2) Let 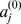 be the minimum gray value of pixels in the above class corresponding to 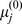, and denote the translation component by 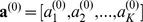.

(3) The relationship among the mean value 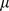, the mode 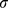 and the translation component 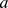 are deduced from the Rayleigh distribution [46] is
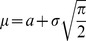
(22)


So the mode 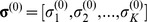 can be computed from Equation (22) as
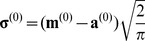
(23)


(4) Let 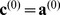 and 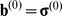.

Step 2: compute the posterior probability.

For 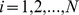 and 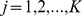, the class-conditional probability density 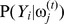 and the prior 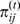 can be computed by Equation (9) and Equation (13), respectively. Then we can compute the posterior probability 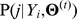 by the Bayesian theorem [24] as
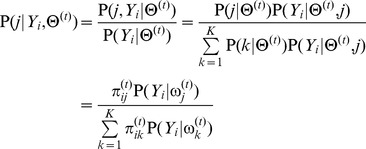
(24)


Step 3: update the parameter set.

By substituting Equation (24) into Equation (17) - Equation (21), Equation (16) can be computed. Then we substitute Equation (16) into Equation (15) in order to update the parameter set from 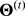 to 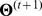.

Step 4: reach the convergence.

After the *t*th iteration, if 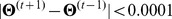, the iteration process can reach the convergence. If not, return to the Step 2 and continue the iteration.

### D Computing the prior of the MRF

The prior of the MRF can be computed as follows. Firstly, the dark region in 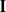 below MIC is located by the results of RMM classification, which contains the acoustic shadowing behind the calcified plaque. In 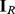, we can compute the mean gray-scale values of pixels from the Class 1 to the Class 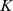, denoted by 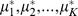, respectively, where the two smallest values 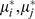 can be found, and the set of the pixels in the two classes corresponding to 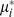 and 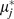 can be considered as the dark region in 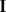, denoted by 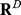. Then we define a binary image 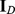, as the same size as 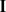, to indicate the location of 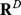, formulated as
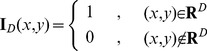
(25)


where 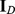 is shown in Figure 2(f). The upper border of 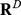 can be defined as the boundary between 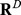 and the other region in 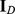, denoted by 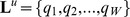, where 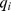 is the row coordinate value of the pixel on the upper border and in the 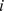
th column of 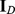. 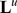 can be computed as
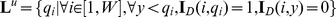
(26)where 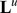 is shown in Figure 2(f). Secondly, the relationship between 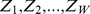 and 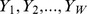 can be influenced by three factors: the distance from the upper border 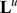 to the edge of the imaging area in IVUS, the distance from 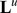 to MIC, and the mean gray-scale value in the region between 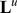 and MIC, which are denoted by 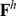, 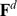, 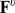, respectively. 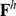, 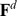, 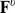 are formulated as
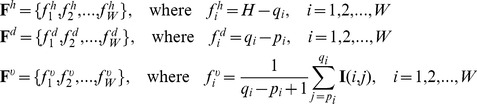
(27)where 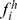, 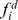, 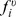 are the values of 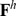, 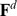, 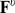 with respect to the 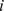
th column of 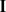, respectively. At last, the prior of the MRF, denoted by 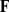, can be quantified as
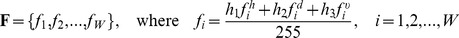
(28)where 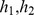, and 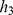 are weights in order to turn 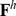, 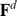 and 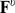 into the same order of magnitude. In our method, 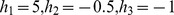.

### E Solving the MRF by the BP algorithm

The BP algorithm can be divided into three parts: the initialization of the MRF and belief propagation messages, the iterative process and the convergence criterion.

(1) Initialization

For 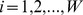, the marginal probabilities of 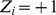 and 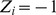 are firstly initialized to be equal, that is, the marginal probabilities are formulated as
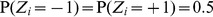
(29)and the belief at 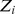 is initialized as the marginal probabilities,
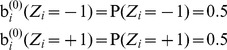
(30)


Then the local evidence 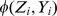 can be represented as the prior of the BP, that is, quantified by 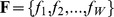 in Equation (28) as
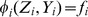
(31)where 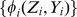 are invariant in the iteration. Next, if 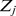 is in the neighborhood of 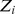, the relationship between 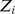 and 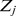 can be represented by the compatible function 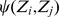, formulated as
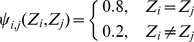
(32)where 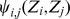 are not updated in the iteration. In addition, the message from 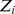 to 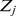, denoted by 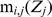, is initialized as
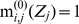
(33)


(2) Iteration

If 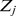 is in the neighborhood of 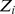, we can compute the message 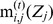 from 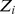 to 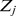 at the *t*th iteration as
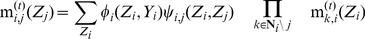
(34)


Then the belief 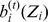 of 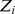 can be computed as
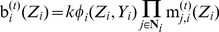
(35)where 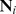 is the neighborhood of 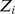. The superscript “(t)” in Equation (34) (35) represent the corresponding variables are in the 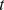
th iteration.

(3) Convergence

The convergence criterion is formulated as follow,
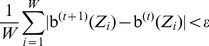
(36)where 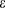 is set to be 0.001 in our method. If Equation (36) is satisfied, the iteration can be terminated.

### F Defining the constraints for refining the calcified plaque

All detected calcified plaques can be combined into a set, denoted by 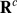. With respect to any calcified plaque in 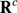 with left border 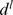 and right border 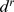, we define five constraints in order to examine whether the calcified plaque is the pseudo calcified plaque or not:

(1) Constraint 1: the coordinates of pixels on MIC between 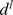 and 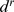 can be represented as 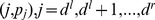. In these 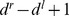 pixels, we compute the rate of the pixels with the gray value larger than the threshold 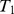, formulated as
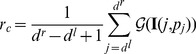
(37)where 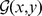 is an indicator function,
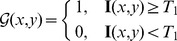
(38)


Then the rate 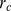 should satisfy
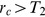
(39)where 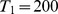 and 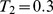 in our method. If Equation (39) is not satisfied, the calcified plaque can be considered pseudo and removed out of 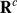.

(2) Constraint 2: the absolute value of the slope of the line between 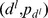 and 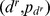 in 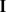 should be smaller than the threshold 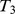, formulated as
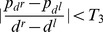
(40)where 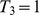 in our method. If Equation (40) is not satisfied, the calcified plaque can be considered pseudo and removed out of 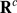.

(3) Constraint 3: the length of the calcified plaque along the angular axis in 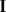 can be represented as 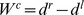, and then the length should be smaller than the threshold 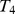, formulated as
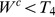
(41)where 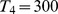 in our method. If Equation (41) is not satisfied, the calcified plaque can be considered pseudo and removed out of 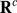.

(4) Constraint 4: two columns 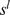 and 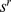 in 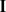 are firstly computed as
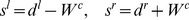
(42)


Then the left region, right region and shadowing region of the calcified plaque can be defined based on 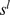 and 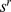, shown in Figure 3(a): (1) the left region is surrounded by four borders in 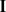: the left border, right border and lower border are three lines between 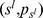 and 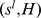, between 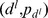 and 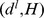, between 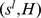 and 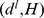, respectively, and the upper border is a part of MIC between 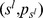 and 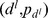; (2) similarly, the right region is surrounded by four borders: the left border, right border and lower border are three lines between 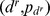 and 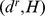, between 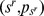 and 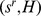, between 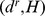 and 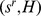, respectively, and the upper border is a part of MIC between 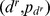 and 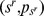; (3) the shadowing region is also surrounded by four borders: the left border, right border and lower border are three lines between 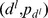 and 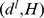, between 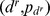 and 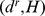, between 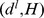 and 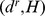, respectively, and the upper border is a part of MIC between 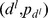 and 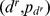. Next, the mean gray values of pixels within the shadowing region, the left region and the right region of the calcified plaque are computed, denoted by 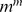, 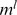 and 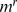, respectively, and the three mean values should satisfy
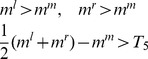
(43)where 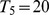 in our method. If Equation (43) is not satisfied, the calcified plaque can be considered pseudo and removed out of 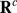.

(5) Constraint 5: with respect to the left border 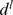 of the calcified plaque, we can firstly find a pixel at 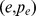 on MIC satisfying 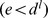


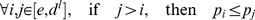
(44)


Similarly, with respect the right border 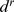, another pixel 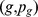 on MIC satisfying 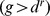


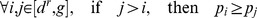
(45)


Then the maximum value between 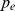 and 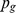 can be found, formulated as
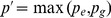
(46)


**Figure 9:**
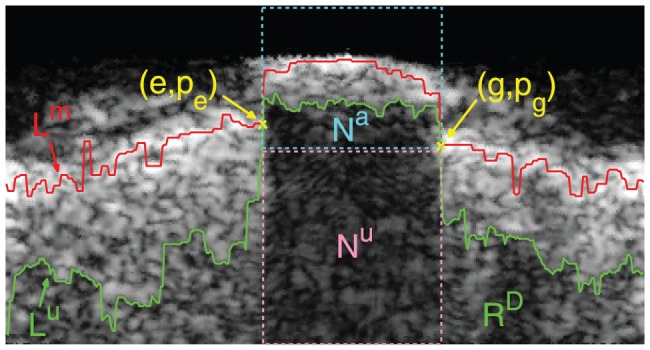
The left and right yellow points represent the points with coordinate 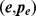 and 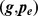, respectively. The red curve and the green curve are 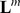 and 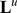, respectively. The region below 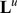 is 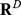. 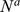 is the number of pixels inside the intersection between the blue dashed rectangle and 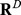. 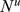 is the number of pixels inside the intersection between the red dashed rectangle and 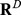.



Next, let 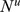 be the number of pixels in the intersection between 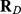 and a rectangle with vertices 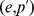, 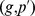, 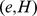, 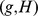, and 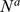 be the number of pixels in the intersection between 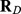 and a rectangle with vertices 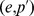, 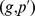, 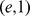, 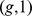. The regions correspond to 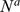 and 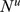 are shown in Figure 9, and the following conditions should be satisfied:
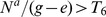
(47)


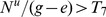
(48)where 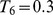 and 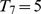 in our method. If Equation (47) and Equation (48) are not both satisfied, then the calcified plaque can be considered pseudo and removed out of 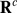.
